# Avian infectious bronchitis virus (AIBV) review by continent

**DOI:** 10.3389/fcimb.2024.1325346

**Published:** 2024-02-05

**Authors:** Saba Rafique, Zohra Jabeen, Treeza Pervaiz, Farooq Rashid, Sisi Luo, Liji Xie, Zhixun Xie

**Affiliations:** ^1^SB Diagnostic Laboratory, Sadiq Poultry Pvt. Ltd., Rawalpindi, Pakistan; ^2^Department of Biotechnology, Guangxi Veterinary Research Institute, Nanning, China; ^3^Guangxi Key Laboratory of Veterinary Biotechnology, Nanning, China; ^4^Key Laboratory of China (Guangxi)-ASEAN Cross-border Animal Disease Prevention and Control, Ministry of Agriculture and Rural Affairs of China, Nanning, China

**Keywords:** AIBV, distribution, continents, prevalence, genotype

## Abstract

Infectious bronchitis virus (IBV) is a positive-sense, single-stranded, enveloped RNA virus responsible for substantial economic losses to the poultry industry worldwide by causing a highly contagious respiratory disease. The virus can spread quickly through contact, contaminated equipment, aerosols, and personal-to-person contact. We highlight the prevalence and geographic distribution of all nine genotypes, as well as the relevant symptoms and economic impact, by extensively analyzing the current literature. Moreover, phylogenetic analysis was performed using Molecular Evolutionary Genetics Analysis (MEGA-6), which provided insights into the global molecular diversity and evolution of IBV strains. This review highlights that IBV genotype I (GI) is prevalent worldwide because sporadic cases have been found on many continents. Conversely, GII was identified as a European strain that subsequently dispersed throughout Europe and South America. GIII and GV are predominant in Australia, with very few reports from Asia. GIV, GVIII, and GIX originate from North America. GIV was found to circulate in Asia, and GVII was identified in Europe and China. Geographically, the GVI-1 lineage is thought to be restricted to Asia. This review highlights that IBV still often arises in commercial chicken flocks despite immunization and biosecurity measures because of the ongoing introduction of novel IBV variants and inadequate cross-protection provided by the presently available vaccines. Consequently, IB consistently jeopardizes the ability of the poultry industry to grow and prosper. Identifying these domains will aid in discerning the pathogenicity and prevalence of IBV genotypes, potentially enhancing disease prevention and management tactics.

## Introduction

Infectious bronchitis, which mostly affects poultry, is a viral disease. It causes substantial economic loss and morbidity in poultry industries worldwide (Cavanagh, 2007; Abozeid et al., 2017). The etiological agents are avian infectious bronchitis virus (AIBV) and avian coronavirus (Carstens and Ball, 2009), which are members of the genus Gamma coronavirus (γCoV), family *Coronaviridae* and order *Nidovirales* (Carstens, 2010; Abro et al., 2012; Durães-Carvalho et al., 2015). The *Coronaviridae* family is divided into four subfamilies, with four genera belonging to the *Coronavirinae* subfamily: alpha coronavirus (αCoV), beta coronavirus (βCoV), gammacoronavirus (γCoV) and delta coronavirus (δCoV). Avian species, including chickens, land fowl, and pheasants, are specifically infected by γCoV and δCoV (Siddell and Snijder, 2014).

**Figure 1:**
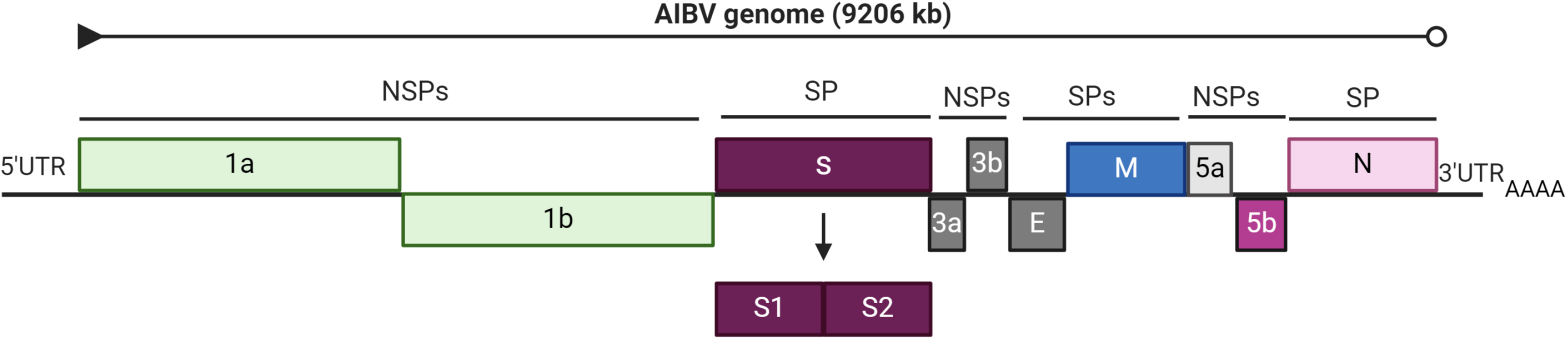
Genome organization of Avian infectious bronchitis virus. The total genome of AIBV is 9206 kb, showing structural proteins (SPs) and non-structural proteins (NSPs). The figure was created with BioRender.com.

The genome of IBV is composed of linear and positive single-stranded RNA strands approximately 27-28 kb long that encode a variety of structural and nonstructural proteins (NSPs). The IBV genome is organized as 5’UTR-ORF 1a/1b-S-3a-3b-E-M-4b-4c-5a-5b-N-6b-3’UTR, with an a-1 frame shift at the junction of ORF 1a/1b, resulting in the synthesis of the 1a and 1b polyproteins, which are subsequently processed to produce individual NSPs responsible for genome replication and transcription (**Figure 1**) (Bhuiyan et al., 2021). Furthermore, these viruses have important structural components, such as the spike (S), membrane (M), envelope (E), and nucleocapsid (N) proteins (Li et al., 2005). Notably, the spike protein, with a molecular weight of 200 kDa, is the largest glycoprotein among these proteins. Previous research has also identified two accessory genes, ORF3 and ORF5, encoding proteins 3a, 3b, 5a, and 5b (Lai and Cavanagh, 1997; Casais et al., 2005). The ORFs, 1a and 1ab are approximately 20 kb in length and constitute approximately two-thirds of the genome. NSPs are a collection of 16 proteins that play indispensable roles in viral replication, RNA synthesis, transcription, RNA proofreading, RNA capping, and host immune response modulation (Ziebuhr, 2005; Chen et al., 2009; Smith and Denison, 2012; Te Velthuis et al., 2012; Kint et al., 2016). The genes for the structural proteins and accessory proteins are ordered in the viral genome as follows.

Gene 2 encodes S protein and is the longest gene in this region and allows the virus to attach to host cells and mediates the fusion of viral and cellular membranes, thus contributing to viral tropism (Kant et al., 1992; Shan et al., 2018). Gene 3 is a polycistronic gene that encodes three proteins, 3a, 3b, and 3c, and is positioned downstream of gene 2. The 3c protein, generally referred to as the E protein, is required for viral particle assembly (Liu et al., 1991), but the 3a and 3b peptides have yet to be identified (Fischer et al., 1998). Gene 4 is another polycistronic gene that encodes three distinct peptides. The first ORF encodes an M protein that is a significant component of the viral envelope. The functions of the two small accessory 4b and 4c peptides are unknown. The M protein contributes to virion structure and may activate a humoral immune response in chickens, but this response is insufficient to prevent clinical signs from appearing (Ignjatovic and Galli, 1994). Gene 5 encodes two tiny accessory peptides, 5a and 5b (Youn et al., 2005; Laconi et al., 2018). Gene 6 encodes the N protein and is the last ORF of the IBV genome. This protein is a nonspecific RNA-binding protein and has numerous roles in the virus’s life cycle (Zúñiga et al., 2010; Kint et al., 2016).

IBV is traditionally thought to be a host-specific respiratory pathogen in poultry, and it replicates at the tracheal mucosa (Amarasinghe et al., 2018). However, the discovery of new IBV genotypes or serotypes has revealed a wide range of tissue tropisms, including in the bursa of Fabricius (Macdonald and McMartin, 1976; Ambali and Jones, 1990), urinary tract (Cumming, 1969; França et al., 2011), reproductive tract (Boroomand et al., 2012) and gastrointestinal tract (Cook and Ambali, 1986; Ambali and Jones, 1990; Liu et al., 2006). IBV is known to replicate in layers of the reproductive tract epithelium, resulting in decreased egg production and defective eggshell formation (Cook et al., 2012). Early infection with the IBV JX181 (GVI-1) strain is highly harmful to laying hens, causing major respiratory problems, irreversible oviduct damage, and growth retardation in the reproductive system (Bo et al., 2022). False layer syndrome, which is associated with cystic oviduct development, cystic left oviducts, signs of vent pecking, ovarian regression, and yolk coelomitis, occurs due to IBV infection in young birds aged 25-28 weeks (Ramsubeik et al., 2023). Cockerel testes can also be infected with IBV (Boltz et al., 2004).

Among all avian species, pheasants are considered the second most common natural host for IBV after poultry. (Gough et al., 1996). Antibodies against IBV have been found in quail, turkeys and free-ranging rockhopper penguins, though it is unknown whether IBV can be isolated from these animals (Karesh et al., 1999). In addition, IBV infects a variety of bird species, particularly those raised near domestic poultry, such as ducks, teals, geese, pigeons, partridges, guinea fowl, peafowl and domestic fowl (Cavanagh et al., 2005). The genome of IBV shares many similarities among various hosts. One virus found in teal and peafowl, for instance, shows 90% to 99% similarity in sequence to IBV (Liu et al., 2005). Incredibly similar nucleotide sequences were found in viruses collected from turkeys, ducks, pheasants, and whooper swans (Hughes et al., 2009). IB was first detected in the United States in 1931 (Schalk and Hawn, 1931). The causative agent was not identified, which frequently caused confusion with other viral and bacterial pathogens that can cause upper respiratory infections in chickens. Until the first report of the Connecticut isolate (Conn) (GI-1), the Massachusetts isolate (Mass) (GI-1) was the sole serotype discovered. The Conn (GI-1) causes identical symptoms but exhibits antigenic variations from the Massachusetts isolate (GI-1) (Jungherr et al., 1956). Notably, this viral pathogen has demonstrated a remarkable capacity for diversification and adaptation, resulting in the identification of numerous unique serotypes in various parts of the globe (Rafique et al., 2018b).

IBV poses no known threat to human health. Neutralizing antibodies have been found in employees who work on commercial chicken farms, though their impact is uncertain (Miller and Yates, 1968). Furthermore, there is currently little evidence to support the idea that IBV may replicate inside a human host; avians can simply transmit IBV infection to chicks mechanically (Kapikian et al., 1969). The continuing processes of genetic recombination and mutation can be attributed to the ongoing emergence of novel IBV variants. Many of these variants show noticeable antigenic differences, which are carefully displayed by cross-neutralization experiments or monoclonal antibody tests. Nonstructural 1ab and accessory proteins, including 3a, 3b, 5a, and 5b, have been reported to be potential tissue tropism regulators as well as pathogenicity determinants. Moreover, the interaction between the S1 and S2 spike subunits of the virus may control the host range and attachment site of IBV. The host immune response and infection route are additional variables that may contribute to tissue tropism (Wang and Hou, 2023).

Diverse IBV strains have been found and documented in various geographical locations. Notably, the Georgia 08 (GA08) strain Genotype 1 and lineage 27 (GI-27) of IBV are the most common and prevalent in the United States (Jackwood and Jordan, 2021). Furthermore, reports of its presence outside the United States have been documented. Similarly, GII-1 (D1466) occurs infrequently outside western Europe (Cook et al., 1999). However, the GI-13 (793B) and GI-19 (QX) variants are largely distributed in Europe, Africa, and Asia but have not been found in the United States and Australia (Tegegne et al., 2020). The S1 spike gene sequence was used to categorize IBV genotypes, resulting in the identification of nine genotypes spanning dozens of lineages.

Two conditions must be met for classification as a new IBV lineage on the basis of the S1 gene: first, well-supported statistical evidence may usually be ascertained using bootstrap or posterior probability values; second, monophyletic clusters consisting of at least three viruses collected from at least two distinct outbreaks may be present, as defined by (Valastro et al., 2016). There must be 13% or greater uncorrected pairwise distances in the nucleotide sequences for IBV lineages to be defined by the abovementioned criteria. Various mutations, including insertions, deletions, point mutations, and recombinations, exist across different strains. As a result of these events, several S1 variants have emerged and evolved (Jackwood, 2012; Hewson et al., 2014). As reported, recombination often occurs during IBV replication, resulting in the formation of chimeric viruses made up of genetic sequences from several viruses (Abro et al., 2012). Thus, the antigenic diversity of IBV is influenced by many factors.

## Genotypic prevalence on continents

**Figure 2:**
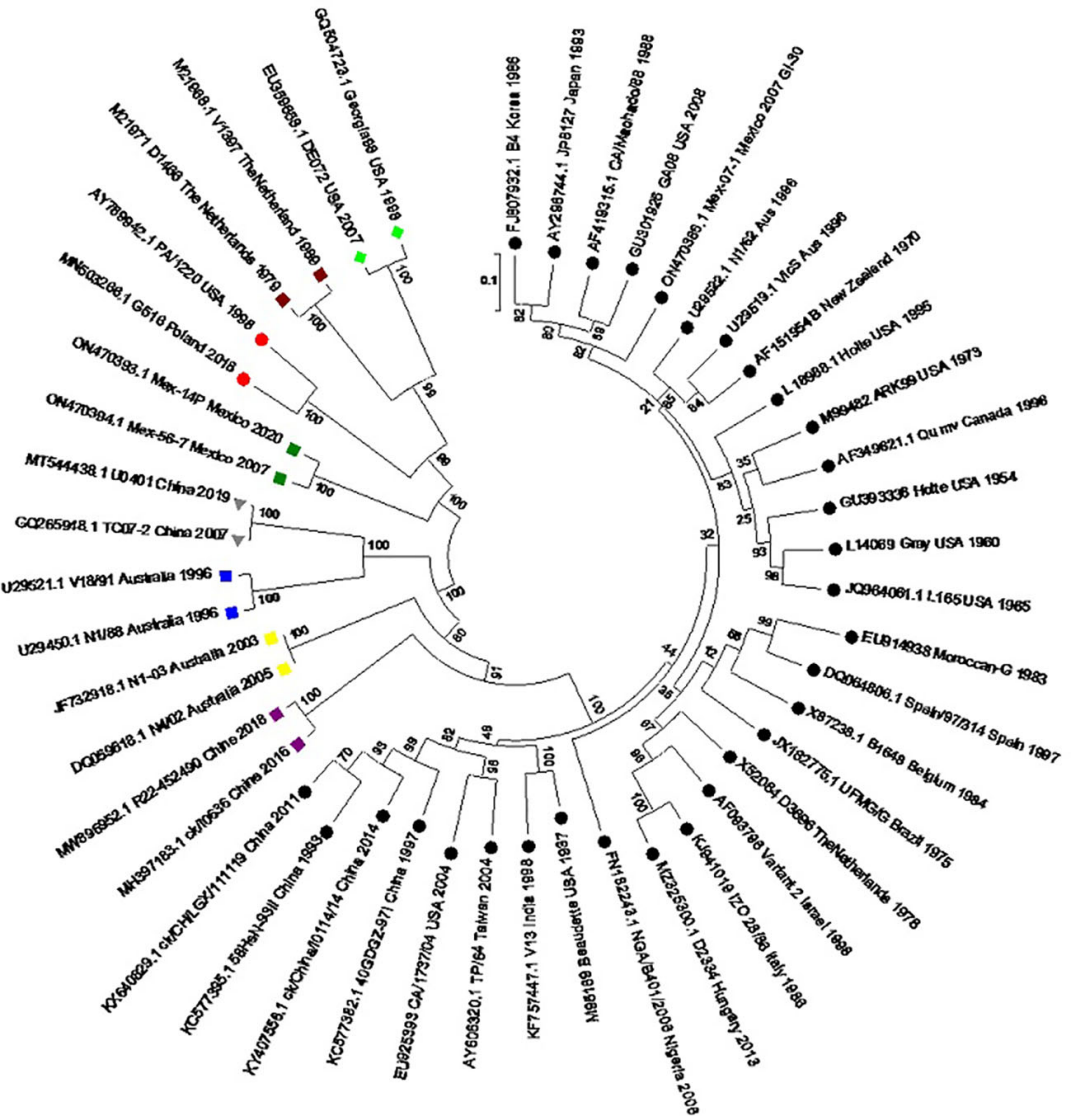
Molecular phylogenetic analysis of the S1 gene of the reference IBV genotypes. A circular tree was created by using the maximum likelihood tree based on the Tamura–Nei substitution model and 1000 bootstrap replicates. Different genotypes are highlighted with different shapes and colors. Black circles: GI lineages (1-31), Meroon square: GII, Blue square: GIII, Green rhombus: GIV, Yellow square: GV, Grey inverted-triangle: GVI, Purple square: GVII, Red circle: GVIII, Green square: GIX.

**Figure 3:**
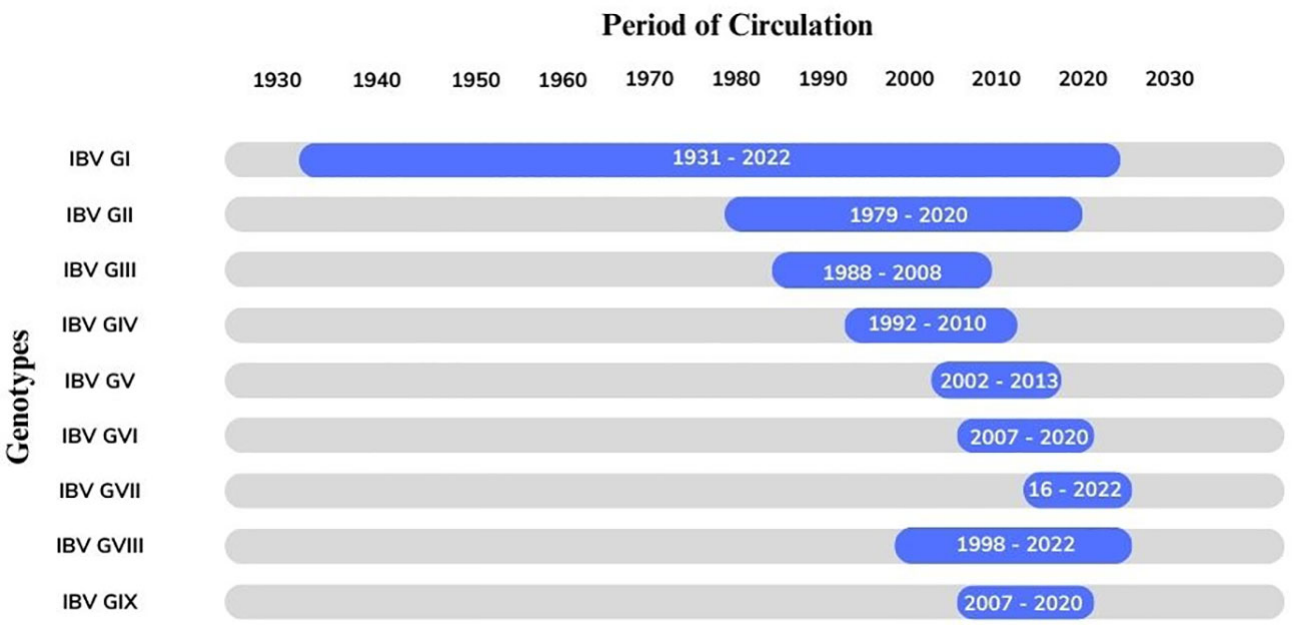
The Gantt chart represents the circulation of the avian infectious bronchitis virus (GI-GIX) genotype throughout the period from 1931–2022.

The sporadic emergence of different genotypes of IBV across continents can be explained individually. The genetic classification of the reported genotypes and lineages was reproduced by using the S1 gene sequence of all IBV genotypes (**Figure 2**). The overall distribution of these genotypes from 1930 to 2022 is also represented using the Ghantt chart (**Figure 3**) and (**Table 1**) to clarify the reported patterns of the genotypic distribution of this virus. The reported patterns are explained in detail in terms of genotype.

**Table 1:** Genotypes of Infectious Bronchitis Virus distributed across continents.

Continent	Reference Strain Name	Prevalent Genotype	References
**America**	Beudette	GI-1	(Valastro et al., 2016; Hassan et al., 2019; Tataje-Lavanda et al., 2019; Najimudeen et al., 2021; Marandino et al., 2022; Ramirez-Nieto et al., 2022; Icochea et al., 2023; Trevisol et al., 2023; Wang and Hou, 2023)
	Holte	GI-2	
	Gray	GI-3	
	Holte	GI-4	
	LI65	GI-8	
	ARK99	GI-9	
	UFMG/G	GI-11	
	B1648	GI-14	
	IZO 28/86	GI-16	
	DMV-1639/11	GI-17	
	Qu_mv	GI-20	
	Varient 2	GI-23	
	CA/1737/04	GI-25	
	GA-08	GI-27	
	Mex-07-1	GI-30	
	DE072	GII	(Icochea et al., 2023)
	GA98	GIV-1	(Gelb et al., 1997; Lee et al., 2001; Mendoza-González et al., 2022)
	DE072	GIV-1	
	MEX-12	GVIII-1	(Mendoza-González et al., 2022)
	Mex-56-7	GIX-1	(Mendoza-González et al., 2022)
**Europe**	Beudette	GI-1	(Gough et al., 2008; Franzo et al., 2014; Valastro et al., 2016; Lisowska et al., 2017; Moreno et al., 2017; Fischer et al., 2020)
	B	GI-10	
	D274	GI-12	
	793B	GI-13	
	B1648	GI-14	
	IZO 28/86	GI-16	
	QX	GI-19	
	Spain/97/314	GI-21	
	Variant 2	GI-23	
	D1466	GII-1	(Domanska-Blicharz et al., 2017; Molenaar et al., 2020)
	D181	GII-2	
	PA/1220/98	GVIII-1	(Domanska-Blicharz et al., 2020)
**Africa**	Beaudette	GI-1	(Ducatez et al., 2009; Toffan et al., 2011; Naguib et al., 2016; Valastro et al., 2016; Zanaty et al., 2016b; Moharam et al., 2020; Bali et al., 2022; Kariithi et al., 2023)
	D3896	GI-12	
	Moroccan-G/83	GI-13	
	B1648	GI-14	
	IZO 28/86	GI-16	
	QX	GI-19	
	Variant 2	GI-23	
	NGA/B401/2006	GI-26	
	D2334/11/2/13/CI	GI-31	
**Asia**	Beaudette	GI-1	(Kumanan et al., 2004; Gaba et al., 2010; Lim et al., 2011; Valastro et al., 2016; Chen et al., 2017; Feng et al., 2017; Rafique et al., 2018b; Lee et al., 2021; Lian et al., 2021; Müştak et al., 2022; Chen et al., 2023; Saleem et al., 2024)
	TP/64	GI-7	
	D274	GI-12	
	PAK 973	GI-13	
	B4	GI-15	
	JP8127	GI-18	
	QX	GI-19	
	40GDGZ-971	GI-22	
	Variant 2	GI-23	
	V13	GI-24	
	LGX/111119	GI-28	
	I0111/14	GI-29	
	DI466	GII-1	(Khaltabadi Farahani et al., 2020)
	K069-01	GIII	(Li et al., 2010)
	T07/02	GIV	(Li et al., 2010)
	TC07-2	GVI-1	(Li et al., 2010; Mase et al., 2010; Jang et al., 2018; Ma et al., 2019; Wang et al., 2022)
	IO636/1	GVII-1	(Ma et al., 2019)
**Australia**	N1/62	GI-5	(Cumming, 1963; Valastro et al., 2016)
	VicS	GI-6	
	N1/88	GIII-1	(Ignjatovic and Galli, 1994; Sapats et al., 1996; Valastro et al., 2016)
	23/88	GIII-2	
	NI/03	GV-1	(Quinteros et al., 2021)

### Genotype I

#### North America

The first case of IBV was reported in central North American states in the spring of the early 1930s; this is an acute, deadly respiratory condition that appeared to be limited to only chicks aged 2 to 20 days. The chicks developed resistance with age, and those older than three weeks were immune. By the early 1970s, multiple distinct IBV variants had been identified, primarily through serological reports (Hitchner et al., 1966; Hitchner et al., 1966; Hopkins, 1974; Cowen and Hitchner, 1975; Johnson and Marquardt, 1976). In North America, the GI-9 and GI-27 and GIV-1 genotypes have been linked to continent-wide disease transmission and persistent viral infections (Valastro et al., 2016; Bande et al., 2017).

Previously, Arkansas (Ark) (GI-9) was the most prevalent IBV strain known in North America, but recently GA08 (GI-27) became a dominant genotype in this region due to high usage of this strain in vaccines (Fields, 1973; Jackwood and Jordan, 2021). Another significant IBV strain in the United States is DMV/1639/11 (GI-17), which was first documented in the United States in 2011 and has significantly affected broilers, breeders and layers from 2014–2015; since then, it has prevailed in certain regions of the US (Jackwood and Jordan, 2021).

The IBV 4/91 strain IBV/Ck/Can/17 038913 (GI-13) was identified from chicken flocks in eastern Canada that had reduced egg production and egg quality from 2011 to 2018. The isolate was assigned to the GI-13 genotype based on comparative genomics and phylogenetic analysis of the S1 gene, and it exhibited varying similarity with different open reading frames (ORFs) of several reference strains. Despite having wide tissue tropism in laying hens, IBV/Ck/Can/17 038913 (GI-13) did not affect egg quality or production at the infection dose used in the current investigation. However, the isolate caused considerable macrophage and T-cell migration along with tissue tropism in the kidney (Najimudeen et al., 2021). Although Ark (lineage GI-9), Holte/Iowa-97 (lineage GI-3), Mass-type (lineage GI-1), and Q1-like serotypes have been identified within commercial flocks in Mexico, a new study indicated that in 2019–2021, 793B (GI–13) and California variant CAV (GI–17)-like strains were found (Kariithi et al., 2022).

The Delmarva (DMV)/1639 variant of IBV, which is a member of GI-17, was linked to numerous nephropathogenic IBV outbreaks that occurred in the Delmarva Peninsula, United States of America (USA), in 2011. IBV strains genetically similar to the DMV/1639 (GI-17) type have been more prevalent in the poultry industry in eastern Canada since 2015. The Canadian DMV/1639 (GI-17) strain has been proven to cause noticeable pathological lesions and tissue tropism in many bodily systems, including the respiratory, reproductive and renal systems (Ali et al., 2022). Another study provided a clear illustration of the distribution of DMV strains, in which five different IBVs were isolated from Ontario, Canada. S gene analysis of these strains revealed high similarity to that of the Delmarva (DMV/1639) (GI-17) strain, and the initial detection of this virus occurred during an IBV epidemic on the Delmarva Peninsula in the USA in 2011. However, whole-genome analysis revealed its relevance to the Conn (GI-1)-type vaccine strain. These isolates were grouped according to the GI-17 genotype (Hassan et al., 2019). Furthermore, a study illustrated the importance of the GI-17 lineage in North American regions**;** specifically, a virus involved in the IBV epidemic that occurred in Costa Rica from May 2016 through mid-2017 was identified and categorized as a Georgia 13-like on the basis of the S1 region of the S gene. The entire genome sequence has a sequence identity with that of the strain from Georgia, USA, of 94.03%, and the least similar sequence identity with that of the strain from China is 86.03%. The Costa Rican isolate, which shows 96.89% similarity with the S1 subunit of the Georgian strain, was identified as belonging to genotype I and lineage 17 (GI-17) (Villalobos-Agüero et al., 2021).

#### South America

In the 1950s, IBV spread to South America, with Brazil having the first mass (GI-1) serotype isolate (de Wit et al., 2011). IBV was recognized as a serious threat to commercial chicken populations by the mid-1980s. New mutations were discovered alongside the Conn (GI-1) and Mass (GI-1) serotypes in both layer and broiler birds (Cubillos et al., 1991).

In South America, the GI genotype has exhibited two distinct lineages, GI-11 and GI-16, which have been in extensive circulation for many years. An entirely South American lineage known as GI-11 arose in Brazil, Uruguay, and Argentina in the 1960s. Conversely, the GI-16 lineage is ubiquitous and consists of two genetic groupings that were previously thought to be independent: Q1 (GI-16) and 624/I (GI-16). The 624/I variant was initially detected in 1993 in Italy and subsequently in Slovenia, Poland, and Russia. Emerging in the late 1970s, the GI-16 lineage first appeared and is now found in the majority of South American states. Retrospective research has shown that it has been prevalent in Italy since the early 1960s, with a substantial drop in disease incidence since the 1990s. Q1 (GI-16) was also found in birds with proventriculitis in China in the 1990s and subsequently in other Asian nations, South America, Europe and the Middle East. Recent sequence analysis of the whole genomes of the Italian 624/I (GI-16) and Q1(GI-16) strains support the idea that these two genetic lineages have a common ancestor (Marandino et al., 2022). The IBV strain known as VFAR-047 was discovered in 2014 in the broiler flock and was referred to as GI-16 in Lima, Peru’s northern area. Examination of the S gene and genome sequencing allowed for the identification of this isolate (Tataje-Lavanda et al., 2019). According to a recent Peruvian study, the circulation of GI-16 has been determined along with the GI-1 (vaccine derived) lineage in the poultry industry of Peru. GI-I is prevalent in tropical areas, and GI-16 is prevalent in coastal areas (Icochea et al., 2023).

According to a report from Trinidad and Tobago, the circulating IBV strain shows more than 20% nucleotide differences from the vaccine strain M41 (GI-1) but 16.7% from the GI-14 lineage. Since the Trinidad and Tobago strains do not cluster with other lineages, they are distinct strains of a completely new lineage (Brown Jordan et al., 2020). Apart from the prevalence of the GI-11 and GI-16 genotypes in South America, the first report of the IBV GI-23 variant was recorded in Brazil in 2022. In 1998, for the first time, the GI-23 variant was found to cause disease in poultry in India. Immediately after, the virus quickly spread to other continents. Among IBV variants, GI-23 strains are significantly pathogenic to the respiratory tract of embryos and specific pathogen-free (SPF) chicks (Trevisol et al., 2023). Another study from Colombia, the country where IBV was initially discovered in 1963, reported the prevalence of GI-1 (Ramirez-Nieto et al., 2022).

In Mexico, during 2007 and 2021, 17 avian infectious bronchitis virus strains were isolated from diseased chickens. Different strains were grouped into different lineages; however, six strains in genotype 1 did not belong to any of the identified lineages. Therefore, after clustering in a well-supported clade, the cells were designated as a new lineage (GI-30) (Mendoza-González et al., 2022).

#### Europe

The Mass (GI-1) serotype of IBVs was considered the only disease-causing IBV until the late 1970s. Dedson & Gough announced in 1971 that other IBV variants had been found in the UK (Dawson and Gough, 1971); subsequently, employees at the Doorn Institute discovered IBVs in mass-vaccinated commercial flocks that belonged to not less than four distinct IBV serotypes linked to disease occurrence (Davelaar et al., 1984). According to a surveillance study conducted in Europe, the most prevalent IBV strains found were Massachusetts (GI-1), QX-like (GI-19), 793B (GI-13), D274 (GI-12), and D1466 (GII-1) (Worthington et al., 2008; Pohjola et al., 2014).

The GI-19 genotype was initially reported in China in the late 1990s and was formerly known as the QX (GI-19) genotype; its high prevalence has made it one of the most common IBV (Liu et al., 2005), after which it spread to other Asian, Middle Eastern and European countries in waves. The GI-19 lineage attracted increased amounts of attention when it was first discovered in the Netherlands between 2003 and 2004. This viral strain was detected in the kidneys and oviducts of infected poultry and was linked to a significant prevalence of false layer syndrome. In Italy, sequencing analysis indicated that the S1 gene shared 99% similarity with the QX (GI-19) strain. In fact, the alignment of the sequence of the hypervariable region of the S1 gene of QX (GI-19) with that of the mass (GI-1) and 4/91 (GI-13) strains was 77.1% and 81%, respectively (Terregino et al., 2008). Later, in the 2000s, the Chinese QX (GI-19) strain was detected for the first time in the United Kingdom (UK). Sequence analysis revealed 96.6% similarity to the original Chinese QX (GI-19) strain and 98.1% similarity to the common European QX-type (GI-19) IBV known as L-1148 (Gough et al., 2008).

Research carried out in Spain and Italy revealed the simultaneous spread of various IBV genotypes. The genotypes that were most prevalent in the two countries in recent years were 793B (GI-13) and QX (GI-19) (Franzo et al., 2014). However, a new IBV variant linked to the XDN variant has emerged (Ji et al., 2011). The Spanish IBV isolates and the XDN strains were found to form a homogenous clade, and both belonged to the QX (GI-19) genotype, while the Italian strains exhibit greater variation in their genomes. The large and homogeneous clade identified in Spain was thought to have descended from a single recombinant parent and then dispersed across the entire nation. In contrast, nine Italian recombinants, which were distinguished by three distinct recombination events and resulted from independent recombination events between the QX (GI-19) and 793B (GI-13) IBV variants, were considered less viable and less suited strains (Moreno et al., 2017).

The GI-23 lineage IBV variant known as Israel strain 2 was initially identified in Israel in 1998 (Meir et al., 2004). The first incidence of IBV VAR2 from the GI-23 genotype was found in Poland in December 2015. The whole-genome analysis of the Polish GI-23 lineage revealed that the mosaic pattern of the viral genome from other IBV strains descended from the QX-like (GI-19), 793B-like (GI-13), and mass-like (GI-1) serotypes. The exact origin of the virus that infects the Polish chicken population is still unknown (Lisowska et al., 2017).

However, a German broiler farm also reported the IBV Israel strain 2. This was the first time that the GI-23 lineage of Middle Eastern origin was isolated in Germany, adding to the increasing concern about the spread of IBV variants across Europe (Fischer et al., 2020). The birds exhibited respiratory and nephropathogenic symptoms. Phylogenetic analysis suggested that the current isolates are members of the GI-23 lineage, indicating that they are most genetically related to Polish variants and the IBV VAR206 (GI-23) vaccine. The possibility of recombination events between Polish GI-23 and German field or vaccine strains is evident given the potential of GI-23 strains to undergo recombination (Zanaty et al., 2016a).

#### Africa

IBV emerged in southern Africa in the early 1980s, resulting in severe disease conditions such as swollen head syndrome (Morley and Thomson, 1984). It was first identified in Mediterranean basin countries. Although IBV is frequently found in both symptomatic and asymptomatic poultry, its effect on the African continent is unknown (Bali et al., 2022).

Although several lineages of IBV have been recovered in Africa, only GI-26 is considered indigenous to the continent and is predominantly composed of strains from North and West Africa (Valastro et al., 2016). Other Eurasian-originating African IBV strains include those from the lineages GI-1 (linked with sporadic IBV outbreaks in many countries), GI-12 (Nigeria), GI-13 (South Africa, Morocco, Ethiopia, Sudan, and Algeria), GI-14 (Nigeria & Cameroon), GI-16 (Nigeria & Côte d’Ivoire), GI-19 (Ghana, Nigeria, South Africa, Zimbabwe, and Algeria), and GI-23 (Nigeria & Egypt), as well as various lineages from Algeria, Tunisia, Libya, and Ethiopia (Valastro et al., 2016; Bande et al., 2017; Moharam et al., 2020).

IBV has not been reported from Kenya or the East or Central African regions up until this point, except Cameroon in Central Africa (Bali et al., 2022). It is possible that the Kenyan strains A374/17 and A376/17 are gastroenteric. S1 gene analysis of this strain revealed its resemblance to GI-23 viruses rather than GI-16 viruses. The four lineages GI-1, GI-13, GI-14, and GI-19 are used to categorize other strains of African IBV. In contrast, the Kenyan isolate A376/17 resembles turkey coronaviruses (TCoVs) from Asia and France more closely than those from North America. However, unlike all other TCoVs that have been studied, TCoVs exhibit distinct differences (Kariithi et al., 2023).

In Ethiopia, sequencing analysis confirmed the presence of 793B genotypes (GI-13). Due to the high frequency of 793B (GI-13) live vaccine administration, these Ethiopian IBV isolates exhibited substantial genetic variation, indicating that they are most likely to be field strains. Only backyard poultry showed positive results, indicating that this type of setting is more conducive to IBV circulation (Tegegne et al., 2020).

There are several different IBV genotypes in Egypt, comprising GI-13, GI-23, GI-1, and GI-16, each with unique genetic and pathogenic characteristics. Based on phylogenetic analysis, the circulating IBV strains in Egypt can be divided into two major groups. The first group consists of strains from the GI-1 lineage that have developed through genetic drift from live attenuated traditional vaccines (Zanaty et al., 2016b). The second group consisted of strains from the GI-23 lineage, including circulating Egyptian variants. According to recombination analysis, a minimum of three distinct IBV strains, 4/91 (GI-13), H120 (GI-1), and QX-type (GI-19), tend to undergo recombination events (Abozeid et al., 2017).

In North African nations such as Tunisia, Egypt, and Morocco, the combination of endemic and classic IBV variants prevails, contributing to nearly all the information on IBV strains in Africa (de Wit et al., 2011). A QX-like (GI-19) IBV strain was recently discovered in Zimbabwe. The IBV strain QX L-1148 (GI-19) from China shows the maximum nucleotide sequence similarity (98.6%) to the S1 gene. Notably, this is the first reported instance of QX-like (GI-19) IBV circulation in Africa (Toffan et al., 2011).

This GI-19 lineage was introduced in 2009, likely as a result of a single introduction event from Germany (Andreopoulou et al., 2019). In Sudan, two IBV strains were identified from commercial broiler farms that exhibited respiratory symptoms and increased mortality. The Chinese IBV strain, the Italian IBV strain, and the 4/91 (GI-13) vaccine strain had the highest nucleotide sequence similarity for the whole genome (93%). The IBV/Ck/Sudan/AR251-15/2014 strain was genetically distinct from formerly defined IBV strains in Africa and worldwide on the basis of S1 gene analysis and was clustered with viruses of the GI-19 lineage (Naguib et al., 2016). Greece has a high incidence of IBV field strains, with 20 or more of the GI-19 lineage (Andreopoulou et al., 2019).

In a study in Africa, the complete genome sequences of five IBV strains from the sub-Saharan region were analyzed. Based on phylogenetic analysis, three (GI-14, GI-16, and GI-19) lineages prevailed in that region. However, it has also been observed that a strain isolated from the Ivory Coast, D2334/11/2/13/CI, belongs to a distinct lineage within the GI-31 genotype (Bali et al., 2022).

A novel IBV genotype known as “IBADAN” has recently been reported. Analysis of the S1 gene sequence indicated that NGA/A116E7/2006 had 24-25% amino acid and nucleotide variation from the nearest strain of a different cluster, whereas ITA/90254/2005 shows 14% nucleotide and 16% amino acid variation from its closest strain. When the whole genomes of NGA/A116E7/2006 and ITA/90254/2005 were analyzed, 10% nucleotide variation was found, with the IBADAN strain exhibiting a high genetic distance from 9.7% to 16.4% when compared to other known IBV sequences. The S1 protein amino acid sequence revealed particular positions shared by NGA/A116E7/2006 and ITA/90254/2005, differentiating them from other strains (Ducatez et al., 2009).

#### Asia

Several lineages, such as GI-7, -15, -18, -22, and -24, and GVI-1, are exclusive to Asia (Valastro et al., 2016). In Japan, IBV strains have been genotyped via incomplete nucleotide sequence information that included the HVR-1 and HVR-2 regions (Mase et al., 2004; Mase et al., 2021a). The strains were divided into seven genotypes based on this classification: JP-I (GI-18), JP-II (GI-7), JP-III (GI-19), JP-IV (GVI-1), Mass (GI-1), 4/91 (GI-13), and Gray (GI-3). The JP/KH/64 strain (GI-18) was identified from chickens exhibiting respiratory symptoms in 1964 in Japan, and it was the first JP-I genotype to be identified in Japan (Mase et al., 2021a).

The complete genome sequence of the primary genotype JP-I (GI-18) strains was retrieved to further understand the distribution of IBV variations in Japan. Phylogenetic analysis of the full coding sequence of the S1 gene revealed that JP/KH/64 is part of the GI-18 lineage and groups in a single category with the lineage prototype strain JP8127, which was discovered in Japan. Because the JP-I (GI-18) genotype has been reported in China, Taiwan, and Japan (12, 13), studying the evolution of IBV in East Asian countries may be aided by this knowledge of the JP/KH/64 strain (Mase et al., 2021b). Another study in Japan determined and studied the entire S1 gene of 61 IBV strains. Except for three strains (JP/Nagasaki/2013, JP/Kochi/2013, and JP/Nagasaki/2016), all strains grouped into the seven genotypes indicated above and were thought to be produced from recombination within the IBV G1-13 and GI-19 S1 genes (Mase et al., 2022). IBV was first characterized in South Korea in 1986 after respiratory signs were detected in poultry. It was initially classified as Korean group I (K-I) and subsequently assigned to the GI-15 lineage (Song et al., 1998). A nephropathogenic form of IBV, currently known as the Korean group II (K-II) (GI-19) subgroup KM91-like variant (Lee et al., 2004), devastated the South Korean chicken industry in 1990. The QX (GI-19) genotype, originally discovered in 1993 in China, is now widespread worldwide and was introduced to South Korea in 2002-2003. Presently, this particular strain is referred to as the QX-like (GI-19) K-II subgroup variant (Lee et al., 2008). Novel variants of the KM91-like (GI-19) and QX-like (GI-19) strains, termed ‘K40/09-like’, appeared in 2009 and have since become the prevalent strains in South Korea (Lim et al., 2011). According to Valastro et al. (2016), by using the IBV S1 gene categorization method, all K-II (GI-19) subgroups (KM91-like, K40/09-like, and QX-like) and QX genotypes belong to the GI-19 lineage. The S1 gene sequences of 60 South Korean IBV isolates were analyzed, and it was discovered that in addition to the GI-15 and GI-19 IBV lineages, five other subgroups of GI-19 cocirculated and expanded in South Korea (Lee et al., 2021). Since its discovery in 1996, Lineage GI-19 (QX-type) has been the most prevalent IBV strain in China 1996 (Feng et al., 2017; Xu et al., 2018) Furthermore, GI-7 (TW-type) and GI-13 (4/91-type) lineages have been recognized to be significant IBV strains in China, with rare infections of GI-1 (Mass-type), GI-9 (Ark-type), and GI-28 (LDT3-type) also reported (Zhao et al., 2016; Lian et al., 2021). The latest studies found 19 IBV variants in clinical specimens collected in China amid January 2021 and June 2022; these included 12 variants of GI-19, three variants of GI-7, and one each of GI-1, GI-9, GI-13, and GI-28. The most common IBV lineages in China according to these studies are GI-19 and GI-7. The progression and dissemination of IBV GI-7 were also detected, and it was proposed that the region of Taiwan might be the source of the IBV lineage GI-7 and that South China has a significant role in IBV transmission (Chen et al., 2023).

Recently, the respiratory type Mass 41 (GI-1) was the most prevalent type of IBV in India (Kumanan et al., 2004). Elankumaran et al. (1999), reported the presence of the IBV variant 793/B by serological testing but did not detect the virus. Bayry et al. (2005), found a single strain of nephropathogenic IBV (PDRC/Pune/Ind/1/00) in India. Later, Gaba et al. (2010), and Sumi et al. (2012), identified and genotyped viruses, confirming the presence of 793/B, an IBV strain. Current investigations revealed that the Mass 41 (GI-1) genotype was present in field isolates (Patel et al., 2015; Parveen et al., 2017; Jakhesara et al., 2018). From 2003 to 2011, Raja et al. (2020), examined 20 IBV field strains from India, three of which were identified as novel IBV genotypes. These authors confirmed that these strains are similar to the GI-24 genetic lineage (Valastro et al., 2016) but not to formerly reported strains (Bande et al., 2017).

The IS-1494 Mahed (variant-2; GI-23) nephropathogenic IBV strain was originally discovered in Iran in December 2015. The strains were grouped with QX (GI-19) and 4/91 (GI-13) according to nucleoprotein gene analysis. However, phylogenetic analysis revealed it to be a chimeric strain (Mousavi et al., 2018).

In Pakistan, there have been few reports of IBV circulation. Ahmed et al. (2007) documented the dissemination of several European IBV variants in addition to the Massachusetts type. Many IBV vaccines were not effective locally, particularly those with D-1466 (GII-1), 4/91 (GI-13), D-274 (GI-12), or Mass-41 (GI-1), indicating the existence of unidentified IBV variants in the field. Rafique et al. (2018b), detected the existence of IBV in a one-year study that monitored the spread of IBV in Pakistan. Their results indicated the presence of mass (GI-1)- and 4/91 (GI-13)-type IBV in 43% and 51%, respectively, of the isolates, but only 5% of the various untyped IBV variants were present. Current analysis also revealed one unique Pak-973 isolate (KX013102_Ck/Rwp/NARC-973/2015_Pakistan). Compared to the 4/91 (GI-13) and mass (GI-1) vaccine strains, the isolate had distinct mutations. Another study by Rafique et al. (2018a), molecularly characterized the field strain KU145467_ NARC/786_Pakistan_2013 (also known as Pak-786) in the same year. The Pak-786 isolate is a member of the GI-13 lineage and includes both vaccine and field strains formerly ascribed to the 793B group (Rafique, 2018). A recent study revealed that IBV-17 of the GI-24 lineage differs from the vaccine strain GI-23, which has been widely used in Pakistan. These variations can result in changes that allow GI-24 lineage viruses to avoid vaccines designed for the GI-23 lineage. It appears that GI-24 has taken over as the main lineage of Pakistani field IBV isolates throughout the past few years (2017-2020) (Saleem et al., 2024).

A ‘HFT-IBV’ variant was discovered in a layer chicken flock in Israel that was routinely vaccinated against 4/91 (GI-13) and H120 (GI-1) variants. The disease was caused by the Israeli variant IS/1494/06, which belongs to the GI-23 lineage. The HFT-IBV isolate shares 97.7% nucleotide sequence similarity with the IS-Var2-like isolates but less than 90% nucleotide sequence similarity with the M41-related (GI-1) (H120, Ma5, M41) and 4/91 (GI-13) (793/B, Moroccan G/83, and CR88) strains. IS-Var2-like genotypes have been assigned to the GI-23 lineage, whereas M41 was assigned to the GI-1 lineage; 4/91-like viruses were assigned to the GI-13 lineage (Öngör et al., 2021). The IS var-2 IBV field isolates recovered from commercial broiler flocks in Turkey from 2014 to 2019 were discovered to be on the same branch as the GI-23 genotype, which has 99% similarity and is one of the most common wild-type clusters in the Middle East (Müştak et al., 2022).

In 2014, three infectious bronchitis virus (IBV) strains were isolated and identified from hens suspected of being infected with IBV in Guangxi Province, China: CoV/ck/China/I0111/14, CoV/ck/China/I0114/14, and CoV/ck/China/I0118/14. S1 sequencing and phylogenetic analysis revealed that the three IBV isolates are genetically distinct from other known IBV types, indicating the presence of a novel genotype (GI-29) (Jiang et al., 2017).

#### Southeast Asia

IBV lineages GI-1, GI-13 and GI-19 are prevalent. According to a study confirmed the presence of various IBV lineages in Thai chicken flocks, along with a novel recombinant IBV variant that originated from the GI-19 and GI-13 lineage viruses (Munyahongse et al., 2020). In another study, variant IBV isolates were categorized into four groups in a study conducted on IBV infection in chickens in Thailand between 2014 and 2016: QX-like IBV (GI-19), Massachusetts (GI-1), 4/91 (GI-13), and a novel variant. QX-like was the most prevalent IBV genotype among these groups in Thailand (Munyahongse et al., 2020). In a recent study, vaccine strains were compared with local common Malaysian IBV strains with two isolates of ACoVs from guinea fowl and jungle fowl. The two isolates from the sample were categorized as genotype I and placed in the GI-13 lineage alongside three other frequently occurring local vaccine strains, namely, CR88, 793B, and 4/91. Molecular characterization revealed homology to the common IBV vaccine strain 4/91 (GI-13) among ACoV isolates from diagnostic cases of jungle fowl (isolate 2015) and guinea fowl (isolate 2016; Besar et al., 2023). In the Vietnamese provinces of Hanoi, Thainguyen, and Haiphong, three strains of IBV, known as VNUA3, VNUA8, and VNUA11, were isolated from sick or infected chickens. The Vietnam isolates belonged to three genotypes: Q1-like (GI-16) (VNUA3), QX-like (GI-19) (VNUA8), and TC07-2-like (GVI-1) (VNUA11). This finding indicates that at least three different IBV genotypes are circulating in North Vietnamese poultry (Le et al., 2019). A study conducted at four farms in Myanmar revealed through phylogenetic analysis of the S1 gene that the IBVs are closely related to the C-78 (GI-18) IBV vaccine strain, while the IBV found at farm 2 was found to be closely related to the GN strain (both of which are categorized as JP-1 (GI-18) types). The isolates from farms 4 and 5 are similar to K446–01 (mass type) (GI-1) and TM86 (JP-2 type (GI-7)), respectively. All of the identified IBV types are extensively distributed, and commercial vaccines targeting their weakened strains are accessible. The JP-1 (GI-18), JP-2 (GI-7), and Mass (GI-1) types of IBV were found to be present in Myanmar poultry farms during this survey (Fujisawa et al., 2019).

#### Australia

In this geographically isolated country, IBV evolved distinctly from the rest of the world (Ignjatovic et al., 2006). Since the early 1960s, several distinct IBV variants have been identified and characterized (Cumming, 1963), and several lineages (GIII-1, GI-6, GV-1, GI-5, and GI-10) have been found to be exclusive to Australia and New Zealand (Valastro et al., 2016).

The traditional IBV variants from Australia have been divided into two distinct lineages, GI-5 and GI-6, which can be identified by the VicS and N1/62 vaccine strains. Surprisingly, both the N1/62 and VicS strains were discovered in 1962, albeit in different parts of Victoria and New South Wales (Cumming, 1963). Although their S1 gene sequences are 83% identical, their distinction into distinct lineages indicates either a unique ancestral origin or major divergence from a parental strain at the time of isolation (Sapats et al., 1996; Mardani et al., 2008; Quinteros et al., 2015).

IBV was not prevalent in New Zealand until the 1970s, when the first case of IB emerged (Lohr, 1976). Notably, strains from New Zealand are included in the GI-5 and GI-6 lineages. New Zealand’s Strain A clustered in the GI-6 lineage (Sapats et al., 1996; Mardani et al., 2008; Quinteros et al., 2015). Six indigenous New Zealand viruses, three of which were identified in the 1970s and the remainder in the 2000s, are part of the GI-10 lineage (McFarlane and Verma, 2008). This IBV strain was identified in the region for the first time in 1967 (Pohl, 1967). According to virus neutralization testing, 4 serotypes (A, B, C, and D) of the virus were identified in New Zealand in 1976; these serotypes differ from those found in other countries (Lohr, 1976). Four new IBV variants, K43, T6, K32, and K87, were discovered later in 2008 in clinically infected flocks. The sequence homology between these strains and the previously mentioned B, C, and D strains is more than 99% (McFarlane and Verma, 2008). Phylogenetic analysis of New Zealand strains confirmed the relevance of these strains to Australian (Vic S) (GI-6) strains rather than European or North American strains (Ignjatovic et al., 2006).

### Genotype II

#### Europe

In the late 1970s, the GII-1 lineage of IBV (also known as D1466 (GII-1) or D212 variation) was identified initially in the Netherlands as an etiological factor associated with egg loss (Davelaar et al., 1984; de Wit et al., 2011). Due to differences between its S1 coding region and those of other European IBV strains, the variant was grouped with the Dutch V1397 strain under the GII genotype (Valastro et al., 2016). Many studies indicate that compared to other IBV strains, this variant has considerably different antigenic and molecular characteristics (Lin and Chen, 2017).

For many years, the D1466 (GII-1) strain was only sporadically recognized, but the findings of a genetic investigation carried out between 2005 and 2006 revealed that this variant was causing additional problems in Western European countries (Worthington et al., 2008). Only a few infections are detected by the D1466 (GII-1) mutation in the UK and France; nevertheless, the disease dynamics are increasing in other European countries. In countries such as Germany, the Netherlands, and Belgium, the prevalence of the D1466-like (GII-1) virus was on average between 3% and 5% in 2005 and increased to 10%, 7%, and 16%, respectively, in 2006. In Poland, the conventional nested RT−PCR technique was used from November 2011 to December 2013. The first D1466 (GII-1) IBV was detected during this time, resulting in 26 positive samples or a prevalence of the variant in 11.7% of the surveyed chicken flocks. Our findings revealed that the prevalence of GII-1 IBV is gradually decreasing in Poland. Certain D1466-positive chicken flocks were declared healthy by healthcare personnel, indicating that the virulence and pathogenicity of the GII-1 strains are not severe (Domanska-Blicharz et al., 2017).

GII-2 (D181) is a novel IBV strain that emerged from an unexpected report in 2017 and became the second most isolated IBV strain among the breeders and layers in the Netherlands in 2018. This strain was also found in Belgian and German samples. According to the entire S1 gene and maximum likelihood analysis, D181 is more closely related to GII-1, commonly known as D1466 (GII-1), than to any other IBV strain, the latter of which shares 90% of the sequence. The remaining 10% are mutations that are distributed throughout the whole S1 gene, and a recombination study provided no evidence that the S1 gene resulted from recombination between D1466 (GII-1) and other IBV strains (Molenaar et al., 2020).

#### South America

The IBV genotype GII lineage was identified in Georgia, USA, in 2000, and its whole genome was submitted to GenBank under accession number AF274435.1 (Icochea et al., 2023).

In an investigation, RT−PCR was employed to identify the IBV D1466 strain of genotype GII-1 (Khaltabadi Farahani et al., 2020). This strategy was validated by studying the spike protein-encoding area in the proprietary S gene of the GII-1 pedigree (pseudo-D1466 strain), which corresponds to IBV(Domanska-Blicharz et al., 2017).

### Genotype III

#### Australia

The GIII-1 lineage originated in 1988 and was given the name Australian subgroup II (Sapats et al., 1996). The IBV isolates Q3/88 (GIII-2) and N1/88 (GIII-1) were identified from these outbreaks (Ignjatovic and McWaters, 1991; Sapats et al., 1996); they are genetically and antigenically different from all formerly identified classical variants, assigned as new or subgroup 2 variants, and were subsequently categorized as the GIII genotype (GIII-1 and GIII-2 lineages, respectively) (Valastro et al., 2016).

#### Asia

Phylogenetic analysis of the 27 identified IBV strains revealed that amino acid residues of the S1 glycoprotein align with the H120 (GI-1) vaccine variant. The isolated viruses were divided into three genotypes based on their genetic origins (genotype I, II and III). Among these isolates, Li et al. (2010) identified these variants at the genotype III level; these included 3 isolates from 2004, 4 from 2006, 1 from 2007, and 6 from 2008 (Li et al., 2010).

### Genotype IV

#### North America

GIV lineage 1 (GIV-1) is the only North American lineage with a unique genotype. This category included (n = 24) vaccinated and field strains identified during 1992 and 2003. The Delaware variant (DE or DE072) (GIV-1) was one of these variants and first discovered in commercial broiler flocks infected with severe respiratory infections in 1992. This difference was attributed to the distinct genotype and novel serotype of this strain compared to the other strains (Gelb et al., 1997; Mondal et al., 2001). The IBV strains that were once known as GA98 (GIV-1) were found to be similar to the DE variants despite having a different serotype and are still considered a part of the same lineage (Lee et al., 2001). It has been proposed that the GA98 (GIV-1) variant emerged as a result of immunological selection triggered by the DE072 (GIV-1) attenuated live vaccine that was administered throughout the country during 1993 (Lee and Jackwood, 2001). Additionally, in 2000, this lineage included viruses that were found in layer flocks and led to decreased egg production (Mondal et al., 2001).

#### Asia

Two previously known Taiwanese IBV isolates evolved into a single genotype (GIV), indicating that the development phenomenon in Taiwan was isolated (Li et al., 2010).

### Genotype V

#### Australia

The GV-1 lineage, known as Australian subgroup III, was reported in 2002; approximately 14 years later, GIII was identified (Ignjatovic et al., 2006). Respiratory and endemic Australian diseases (4 and 7 variants, respectively) have been characterized in GV lineages. The variants Q1/13 and V1/07 were isolated from broilers with respiratory symptoms in Victoria and Queensland in 2007 and 2013, respectively. These results are identical to those for N1/03 at the genetic level, indicating increased geographic dissemination of genotype GV strains (Hewson et al., 2009).

### Genotype VI

#### Asia

A GVI-1 strain was initially discovered in Guangxi Province, China, in 2007. The TC07-2 strain was shown to be significantly evolutionarily distant from six other main genotypes (Li et al., 2010). GVI was subsequently isolated in South Korea and Japan, among other Asian nations (Mase et al., 2010; Jang et al., 2018). The respiratory tract tropism of GVI-1 strains may be attributed to extensive recombination of gene 3 with the S gene (Ren et al., 2019). Nonetheless, not all GVI-1 variants descended from a single common ancestor.

A variant of IBV GVI is a newly found strain that is not particularly infectious to poultry, but coinfection with an epidemic variant may occur and harm China’s poultry industry. In China, IBV genotype VI (GVI-1) was identified in two separate studies from 2019 to 2020 (Wang et al., 2022). A comparison of the whole genomes of two IBV variant strains in the present study with those of other genotype variants indicated only minor similarities in the 5a, 5b, M, and N genes, with few previously identified GVI-1 variants, but greater similarities with the GI-19, GI-22, and GVII-1 genotype variants. The S gene of GVI IBV was substantially dissimilar to the S genes of the QX (GI-19) and YN (GI-22) strains (Sun et al., 2021).

Furthermore, genotype VI was produced by three classical American strains and one Japanese strain (GI-18), and the isolated TC07-2 (GVI-1) and published DE/072/92 (GIV-1) strains had the greatest evolutionary distances to all six major genotypes, but their significance was unclear (Li et al., 2010). GVI-I was isolated from Japan, including Ibaraki/168-1/2009, JP/Chiba/2010, and JP/Kagoshima-3/2014 and shown to cause clinical symptoms such as a reduction in egg production, nephritis and respiratory problems, respectively (Mase et al., 2022).

Recently, the circulation of the GVI-1 lineage, which was geographically assumed to be contained in Asia, was detected in research in Colombia (Ramirez-Nieto et al., 2022).

### Genotype VII

In addition, another novel genotype emerged from China in the late 2020s and grouped as GVII from the I0636/1 isolate and the GX-NN130021 reference strain because it did not resemble other established lineages. In a study, an in-depth comparison and phylogenetic analysis of 74 complete sequences on the basis of the S gene were carried out that involved 73 representatives from each lineage and genotype along with the I0636/1 strain. Additionally, within the S gene of GI-18, at least two recombination sites are replaced with an unidentified sequence that most likely originated from another IBV strain. As a result, a new serotype with limited respiratory tract tropism in poultry emerged (Ma et al., 2019).

### Genotype VIII

#### Europe

A novel IBV variant was discovered in Poland. This variant differs from previously discovered viruses and is closely related to the North American isolate PA/1220/98. The variant was identified as a distinct isolate on the basis of the S1 coding region and shows homology to other recognized GVII IBV genotypes. This lineage was categorized as distinct within the novel GVIII genotype using the standard criteria for designating a novel IBV genotype or lineage. The nucleotide identity of this strain with any known IBV genotype ranges from 52.7 to 58.1%, with maximum identity (81.4%) with the North American variant. This novel strain was subsequently identified in three other flocks of chickens with poor egg production. Notably, the virus has not yet been found in broilers (Domanska-Blicharz et al., 2020).

#### North America

The newly identified genotype GVIII was found in two Mexican samples closely related to unique Mexican strains (UNAM-97/AF288467) (Valastro et al., 2016). However, GVIII-1 exhibits very little intra-lineage variation, with only a 2% difference in nucleotides and a 3% difference in amino acids. The evidence supporting their classification as a new genotype includes their distinct isolation in the flock and clustering within the phylogenetic tree; a significant divergence of 28% in nucleotides and 45% in amino acids from the nearest GIV-1; and 30 different amino acid alterations. The nucleotide and amino acid sequences of IBV genotypes vary by more than 29% (Mendoza-González et al., 2022).

### Genotype IX

Another genotype that emerged in North America in North America is called GIX-1, and distinct clustering of the two Mexican isolates was observed on the phylogenetic tree. The samples were collected 13 years ago from different parts of the country. With 35% nucleotide variation and almost 50% amino acid variation from the closest genotype, GVII-1, this novel genotype has diverged significantly. The same lineage also exhibits a 6% divergence in nucleotides and a 10% in amino acids. It contains 24 distinct residues and has two distinct amino acid insertions (Mendoza-González et al., 2022).

## Pathogenicity of IBV strain

Diverse genotypes are thoroughly documented alongside their respective pathogenicity indexes and tissue tropism in the (**Table 2**) provided below. The pathogenicity indexes reveal diversity even within identical genotypes, highlighting the complex nature of strain-specific variations. Furthermore, a careful examination reveals that tissue tropism contradicts a one-to-one link with any specific lineage, highlighting the complexities of host-pathogen interactions. This in-depth investigation sheds light on the complex interactions between genotypes, pathogenicity, and tissue tropism, setting the framework for a more sophisticated understanding of microbial dynamics.

**Table 2:** Tissue tropism and pathogenicity of the Infectious Bronchitis Strains.

Genotype	Lineage	IBV Strain	Pathogenicity	Tissue Tropism/Viral Distribution	References
**GI**	GI-1	IBVPRO3/Mass	N.D. (Pathogenic but level is not defined)	Reproductive, Kidneys	(Pereira et al., 2019)
	GI-5	N1/62	High	Kidneys	(Quinteros et al., 2022)
	GI-6	Q1/73	Moderate	Kidneys	(Quinteros et al., 2022)
	GI-6	Vic/S	Low	Kidney, Respiratory, Reproductive	(Quinteros et al., 2022)
	GI-6	Q1/65	High	Kidneys	(Quinteros et al., 2022)
	GI-7	TW-Like	High	Trachea, Lungs, Kidneys, and Bursa of Fabricius	(Yan et al., 2016)
	GI-17	DMV/1639	High	Respiratory, Kidney, Reproductive	(Hassan et al., 2019)
	GI-19	QX	High	Proventriculus, Respiratory, Kidney, Reproductive	(Bo et al., 2022)
	GI-22	YN	High	Reproductive	(Zhong et al., 2016)
	GI-23	EG/1212B-2012	High	Respiratory, Kidney	(Zanaty et al., 2016a)
	GI-28	LGX/111119	High	Proventriculus, Respiratory, Kidney	(Chen et al., 2017)
	GI-29	I0111/14	N.D.	Respiratory, Kidney	(Ma et al., 2019)
	G1-31	D2334/11/2/13/CI	N.D.	caecal tonsil	(Bali et al., 2022)
**GII**	GII-1	D1466	Low-Moderate	Respiratory, Kidney, Reproductive	(Khaltabadi Farahani et al., 2020)
**GIII**	GIII-1	V6/92	Low	N.D.	(Quinteros et al., 2022)
	GIII-1	N1/08	Low	Respiratory	(Quinteros et al., 2022)
**GVI**	GVI-1	JX181	High	Respiratory, Reproductive, spleen, Bursa of Fabricius	(Bo et al., 2022)
**GVII**	GVII-1	I0636/16	Low	Respiratory, Kidney	(Ma et al., 2019)
**GVIII**	GVIII-1	PA/1220/08	N.D.	Respiratory, Kidney	(Domanska-Blicharz et al., 2020)
**NOVEL**	N.D.	CK/CH/GX/202109	High	Respiratory, Kidneys, Bursa of Fabricius, Proventriculus, Gizzard, Ileum, Jejunum, and Rectum	(Wang and Hou, 2023)

N.D, Not Done.

IBV has been linked to a number of clinical symptoms in its host, the domestic chicken. The virus appears to reach host cells via viropexis after initially replicating in the upper respiratory tract (Patterson and Bingham, 1976). Tissue tropism varies between strains, although the reason for this variation is unknown. Variations in IBV tissue tropism contribute to differences in clinical symptoms in infected birds. In general, these distinctions allow viruses to be classified as proventriculus, respiratory, reproductive, or nephropathogenic based on the major clinical presentations (Quinteros et al., 2022). On the basis of pathogenicity, IBV characterized as low moderate, and high pathogenic. These conditions can also have turned vice versa based on secondary and opportunistic microorganism infection. Nephropathogenic IBV strains induce nephritis in hens and are the most pathogenic IBVs, having mortality more than respiratory and reproductive strains (Chen et al., 1996). Proventriculitis causes ruffled feathers and respiratory symptoms such as tracheal rales, nasal discharge, sneezing, and coughing in birds. Ulcers and hemorrhages were found in the proventriculus papillae later on, and the condition is serious (Yu et al., 2001).

## Prevention and control of avian infectious bronchitis virus

To control infectious diseases on modern poultry farms, biosecurity measures, and a productive management system are essential. For the avian influenza virus, this concept depends solely on having appropriate knowledge concerning the variables that affect viral transmission (Boender et al., 2007; Singh et al., 2018). Consequently, there is a dire need to thoroughly analyze the epidemiological factors responsible for the transmission of IBV, particularly the determinants of spread (Franzo et al., 2020; Najimudeen et al., 2020).

Vaccination is still the most effective way to manage IBV infection, despite its limitations, which include serious adverse effects of vaccination in young birds, the need for periodic vaccine replacement due to viral mutation, and the likelihood of viral recombination (Franzo et al., 2016; Ji et al., 2020). Effective and meticulously administered vaccines can reduce the viral load, rate of infection, and occurrence of clinical symptoms (Franzo et al., 2016). Several factors influence the strength and period of response to vaccination include chick age, vaccine immunogenicity, field strain virulence, vaccine administration strategy, extent of maternal immunity, and the time frame between vaccination and challenge. Vaccinated chickens may remain immune for several months, and in the case of broilers, this immunity may persist throughout their lifetime (Bijlenga et al., 2004). The majority of commercial chicken flocks are currently immunized against IBV. The IBV immunization protocol may change based on the type of vaccine used and the particular circumstances of the poultry farm. To maintain immunity, chicks are vaccinated throughout their life beginning at one day old. Booster agents can be administered at 7–10 days, 3–4 weeks, and then every 5–6 weeks afterward. The idea of a protectotype has become more widely accepted for regulating IBV due to its variants circulating worldwide.

Numerous vaccination strategies have been designed to manufacture IBV vaccines that are effective. However, the complex immune protection process prevents the extensive use of new vaccine approaches, which are still in the laboratory research stage (Kapczynski et al., 2003; Yang et al., 2009; Bande et al., 2015). The process of developing in ovo vaccination is also at the research stage. The vaccine will be based on the type of IBV strain and will not kill the embryos (Tarpey et al., 2006). The market offers a variety of IBV vaccines, which might differ in nature and vaccination strains based on local isolates and recombination in variants isolated from various countries with distinct laws and regulations. Heterologous IBV vaccinations effectively provide immunization against the 793B-type (GI-13) variant, which was previously proven to persist with live attenuated IBV vaccines and to be effective against the QX (GI-19) and Italy 02 (GI-21) strains (Cook et al., 1999). In a study in Korea, it was demonstrated that the K2 vaccine may be more potent for preventing and controlling novel IBV recombinants and variants that are spreading (Shirvani and Samal, 2020). To manage IBV in China, H120 (GI-1) vaccination is frequently used in conjunction with the indigenous FNO-55 (GI-13, 4/91-like), QXL87 (GI-19, QX-like), or LDT3-A (GI-28, YN-like) variants.

For serotypes, including Arkansas (GI-9), Massachusetts (GI-1), and Conn (GI-1), improved live vaccines and killed oil-based emulsions are available in North America. The Georgia 98 (GIV-1) and California (GI-17) strain vaccines were obtained from the USA. The vaccinations, designated “Holland variants” D274 (GI-12) and D1466 (GII-1), are generally produced in Europe. Conversely, vaccines based on the H120 (GI-1) strain are being used throughout Europe.

The degree of immunity may rely on regional sources to produce varying levels of immunity and an unusual ability to cross-protect against a few heterologous IBV strains Florida (GI-1) and JMK (GI-3) in the U.S. Overall, combining the IBV 4/91 (GI-13) and Ma5 (GI-1) variant vaccines may provide excellent protection against heterologous IBV strains. Although QX-type (GI-19) live vaccines have been developed in Europe, their use is restricted (Cook et al., 2012). A new generation of IBV vaccines against the regionally dominant D274 (GI-12) variant has been produced for future layer and breeding stocks.

One study demonstrated that the type of tissue, inoculation route and vaccine strain all affect the pattern of IBV replication. The distribution and elimination of the vaccine viruses for Massachusetts (GI-1) and 793B (GI-13) were slower when the viruses were administered by drinking water (DW) than when they were administered via the oculonasal (ON) route. Both vaccines were able to induce similar levels of mucosal immunity when administered via the ON route. Regardless of the vaccination technique, the Mass IBV vaccine induces cellular immunity at comparable levels. The 793B vaccine produced noticeably greater levels of humoral immunity when administered via the ON or DW route (Al-Rasheed et al., 2021). In a study, birds were given bivalent live attenuated IB vaccines containing the Mass and Conn serotypes at intervals of two, five, nine, and fourteen weeks after they were first primed with a monovalent live attenuated IB vaccine (mass serotype) at one day old. There was no apparent difference in the ability of the two vaccination regimens to protect laying hens against mass IBV challenge. These findings suggest that the vaccine strain may have a greater level of protection when faced with homologous IBV strains (Ali et al., 2023). The probability of postvaccination challenges is infrequent, but postvaccination challenges may lead to reversion to virulence in immunocompromised or unvaccinated chickens, which eventually causes significant mortality and the intermittent spread of IBV (Hiscox et al., 2001; Bhuiyan et al., 2021). The inability of birds to generate an adequate immune response after vaccination is the cause of vaccine failure (Bosha and Nongo, 2012). It is noteworthy that vaccination techniques are subject to change over time in response to the introduction of novel IBV genotypes and improvements in vaccine technology. Furthermore, local elements influence vaccination programs differently across different regions. These include disease prevalence, farm size, biosecurity measures so on.

This review emphasizes the critical relevance of understanding genotypic variations to implement effective control measures. The identification of region-specific genotypes provides poultry stakeholders with customized vaccination, biosecurity, and management measures. With the ever-changing IBV landscape, the incorporation of genotypic information into control systems has emerged as a critical tool. This knowledge synthesis not only improves our understanding of IBV epidemiology but also allows for the creation of customized therapies to reduce the impact of this economically significant poultry virus. This review, in essence, serves as a foundation for expanding the understanding and control of IBV, hence encouraging sustainable and resilient poultry production systems worldwide.

## Author contributions

SR: Writing – original draft, Writing – review & editing. ZJ: Writing – review & editing. TP: Writing – review & editing. FR: Writing – review & editing. SL: Investigation, Writing – review & editing. LX: Formal analysis, Writing – review & editing. ZX: Conceptualization, Funding acquisition, Resources, Supervision, Validation, Writing – review & editing.
